# Long‐term efficacy of venous sinus stenting in the treatment of idiopathic intracranial hypertension

**DOI:** 10.1111/cns.14356

**Published:** 2023-07-20

**Authors:** Hui Cheng, Haidi Jin, Yongjun Hu, Lijiang Chen, Zhicai Chen, Genlong Zhong

**Affiliations:** ^1^ Department of Neurology, School of Medicine, Sir Run Run Shaw Hospital Zhejiang University Hangzhou China; ^2^ Department of Neurology, School of Medicine, The Second Affiliated Hospital Zhejiang University Hangzhou China; ^3^ Department of Neurology The Sixth Affiliated Hospital of Wenzhou Medical University Lishui China

**Keywords:** intracranial pressure, idiopathic intracranial hypertension, papilledema, sinus stenosis, stent

## Abstract

**Backgrounds:**

Previous studies have suggested that cerebral dural sinus stenosis could be a possible underlying cause of idiopathic intracranial hypertension (IIH). Venous sinus stenting (VSS) has emerged as a potential alternative for treating IIH related to dural sinus stenosis. However, most of the documented studies have been conducted in Western countries. In this study, we present the results of 16 Chinese IIH patients who underwent VSS treatment in our single center.

**Methods:**

We prospectively collected angiographic and manometric data from IIH patients who underwent angioplasty/stenting. All patients had confirmed dural sinus stenosis and had failed maximal medical therapy (MMT). Demographic, clinical, and radiological presentation, as well as long‐term follow‐up outcomes were collected retrospectively.

**Results:**

A total of 16 patients who underwent VSS were enrolled in the present study. Demographic data revealed a mean age of 40 (range 20–55), with 69% (11/16) being female, and a mean body mass index (BMI) of 27.05 (range 19.18–38.04) kg/m^2^. All patients presented with papilledema and visual disturbances. During a median follow‐up period of 47.5 months, 93.75% (15/16) of patients reported improvement in symptoms, although only 37.5% (6/16) experienced complete resolution. Headaches, blurred vision, and amaurosis related to increased pressure improved in 100% (8/8), 81.25% (13/16), and 75% (3/4) of patients, respectively. However, one patient suffered cerebral infarction and secondary epilepsy soon after VSS, and another patient had recurrence of symptoms due to stent wall thrombosis 2 years later.

**Conclusions:**

The significance of venous sinus stenosis in the development of IIH may be undervalued. Our study, based on a Chinese case series, affirms the long‐term safety and effectiveness of VSS in treating IIH patients with relatively lower BMI than those from Western countries.

## Graphical abstract

Venous sinus stenosis is an underlying cause of idiopathic intracranial hypertension (IIH), which is characterized by elevated intracranial pressure (ICP) and related symptoms. Our study demonstrated that venous sinus stenting (VSS) could effectively resolve the stenosis, decrease the elevated ICP, and improve the outcomes of vision and headache.

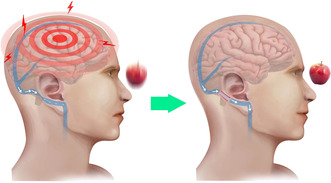

## 1 INTRODUCTION

Idiopathic intracranial hypertension (IIH) is a condition characterized by elevated intracranial pressure (ICP) of unknown origin, without the presence of an intracranial mass, ventricular enlargement, or evidence of venous sinus thrombosis.^1^ The annual incidence of this condition is low, with a rate of 0.5–2 per 100,000 people in the general population. However, it is much more prevalent among young, obese women of childbearing age, with an incidence that is almost twenty times higher than the general population.^2^ The underlying causes of IIH are still largely unknown, and recent studies have focused on the role of cerebral venous outflow obstruction in its pathophysiology. Venous sinus stenosis is a significant and treatable cause of this type of obstruction and the resulting elevated ICP.^3^

Venous sinus stenting (VSS) is a relatively new procedure used to treat IIH and has shown promising results in terms of improved vision and headache outcomes.^4^ However, the long‐term safety and efficacy of this procedure have not been extensively investigated, particularly in patients from Asian countries. This paper reports our experience of 16 Chinese patients with a median follow‐up period of 47.5 months in a single center.

## 2 PATIENTS AND METHODS

### 2.1 Study subjects

We conducted a retrospective study to review the data of patients who underwent VSS for IIH between December 2016 and December 2021. All patients underwent pre‐ and poststent pressure measurement and funduscopy examination. The collected data included demographics, clinical manifestations, peri‐procedural complications, and outcomes of long‐term clinical follow‐up. All patients had failed maximal medical therapy and had worsening symptoms, despite the use of drugs (methazolamide, topiramate furosemide, mannitol, etc.) at maximum tolerable doses, as well as weight loss and repeated lumbar punctures. The Modified Frisen score,^5^ assessed by an ophthalmologist expert who was blinded to the patients' conditions, was employed to grade the papilledema based on funduscopy images. The severity of papilledema was classified into six stages: Stage 0: Normal optic disc or not a disc but no edema/swelling; Stage I: Minimal; Stage II: Low degree; Stage III: Moderate; Stage IV: Marked; Stage V: Severe. The 6‐item Headache Impact Test (HIT‐6) score was used to evaluate the extent of headache‐related disability among all patients.^6^ The total score ranges from 36 to 78, where a higher score indicates a greater impact of headache on the daily life of patients (little or no impact: 36–49, moderate impact: 50–55, substantial impact: 56–59, severe impact: 60–78).

### 2.2 Ethics statement

All clinical investigation has been conducted according to the principles expressed in the Declaration of Helsinki. All subjects had given written informed consent prior to the study, and the protocols had been approved by the local Ethics Committee.

### 2.3 Pressures and peri‐procedural data

Data collected during the procedure included the side of stenosis, balloon and/or stent used, procedural complications, angiographic changes, postprocedural complications, and 24‐h postprocedural clinical outcomes. Angiographic data included the location and degree of stenosis, as well as the pressure gradient across the stenotic area before treatment. Venous pressures were measured in the superior sagittal, transverse, and sigmoid sinuses.

### 2.4 Intervention

The procedures were carried out under general anesthesia. Dual antiplatelet therapy (aspirin 100 mg plus clopidogrel 75 mg) was initiated at least 5 days prior to the procedure. Access to the femoral vein was achieved and a 6 or 8 French sheath was positioned in the jugular bulb over a wire. To raise the activated clotting time over 250 s, intravenous heparin was given. After gaining access, a heparin bolus of 3000 units was administered, followed by a continuous infusion of 1000 units per hour throughout the procedure. A microcatheter, situated within the sheath, was advanced over a 0.014‐inch guidewire into the superior sagittal sinus. Pressures were measured at various sites, including the superior sagittal sinus, torcular, prestenotic transverse sinus segment, and poststenotic transverse sinus or sigmoid sinus, and a pressure gradient was obtained across the stenotic area. The patient then underwent general anesthesia. The microcatheter was exchanged for a balloon angioplasty catheter, positioned across the stenosis, and dilated. The decision to perform balloon dilation is determined by the surgeon's discretion, as it is commonly deemed an unnecessary procedure for VSS in cases of IIH. After balloon angioplasty, a stiff 0.018‐inch guidewire was exchanged into the superior sagittal sinus, and a self‐expanding stent was deployed across the stenotic segment. Following the placement of the stent, the microcatheter was advanced through it to the level of the torcular, and the trans‐stenosis gradient was measured. Dual antiplatelet therapy was continued for 3 to 6 months after stenting, and then a single antiplatelet agent (either aspirin 100 mg or clopidogrel 75 mg) was prescribed for lifelong if no contradiction was present.

## 3 RESULTS

### 3.1 Demographic and presentation

A total of 16 patients, consisting of 5 males and 11 females with a mean age of 40 years (range 20–55), underwent 16 procedures. The average BMI of the patients was 27.05 kg/m^2^ (range 19.18–38.04) and 50% of them were classified as overweight or obese (BMI ≥25). All patients experienced papilledema and visual disturbances, with half (8 out of 16) reporting headache as a symptom. The visual disturbances included symptoms such as amaurosis, decreased visual acuity, or visual field cuts. Pulsatile tinnitus was observed in two patients (12.5%, 2 out of 16). One patient had undergone ventriculoperitoneal shunt (VPS) before being referred for endovascular treatment. The median duration of symptoms lasted for 2.5 months (ranged 0.23–12) before receiving VSS.

The presence of dural sinus stenosis was confirmed in all patients prior to the procedure using either contrast‐enhanced magnetic resonance venogram (MRV) or computed tomography Venogram (CTV). In 56.25% (9 /16) of the cases, the stenosis was located in the transverse sinus, while in 50% (8/16), it was located at the junction of transverse and sigmoid sinuses. Of the 16 patients, 100% (16 /16) had stenosis on the right side, and 56.25% (9 /16) had bilateral stenosis. All patients with unilateral transverse sinus stenosis were accompanied by contralateral dysplasia (Table 1).

**TABLE 1:** Clinical and ophthalmological status preprocedure.

Patient	Age/Sex	Duration of symptoms (month)	BMI	Stenosis location	Major symptoms				CSF pressure (mmH_2_O)	Pressure gradient (mmHg)
					Headache (HIT‐6)	Papilledema (Frisen score)	Other visual symptoms	Pulsatile Tinnitus		
1	52/M	7	26.08	R/L transverse sinus	51	Stage III	Yes	No	340	24
2	53/F	1	24.65	R/L transverse sinus	36	Stage IV	Yes	No	320	14
3	54/F	6	24.16	R transverse sinus	36	Stage III	Yes	No	310	32
4	52/F	7	29.04	R transverse sinus L tran/sig Junction	36	NA	Yes	No	390	26
5	55/M	12	27.04	R/Ltran/sig Junction	76	Stage IV	Yes	No	410	37
6	28/F	1	31.22	R tran/sig Junction	66	Stage IV	Yes	Yes	400	26
7	50/F	6	27.12	R/L tran/sig Junction	36	Stage II	Yes	No	300	22
8	20/F	0.67	19.18	R tran/sig Junction	78	Stage III	Yes	No	400	36
9	34/F	3	38.04	R transverse sinus	63	Stage II	Yes	No	330	17
10	45/M	2	24.51	R transverse sinus	36	Stage III	Yes	Yes	250	20
11	45/F	2	23.24	R/L tran/sig Junction	36	Stage IV	Yes	No	330	28
12	25/M	7	24.44	R/L tran/sig Junction	71	NA	Yes	No	400	18
13	25/F	2	29.94	R/L tran/sig Junction	78	Stage IV	Yes	No	340	10
14	22/F	2	35.43	R transverse sinus	52	Stage IV	Yes	No	400	48
15	54/F	5	24.22	R/L transverse sinus	36	NA	Yes	No	310	17
16	21/M	0.23	24.51	R/L transverse sinus	36	Stage III	Yes	No	400	20

Abbreviations: BMI, body mass index; CSF, cerebrospinal fluid; F, female; HIT‐6, Headache Impact Test‐6; L, left; M, male; NA, not available; R, right; tran/sig, transverse/sigmoid.

### 3.2 Intervention and complications

All 16 patients underwent VSS treatment for dural venous sinus stenosis under general anesthesia. All stents were placed in the right lateral sinus. The average prestenting pressure gradient was measured at 24.69 mmHg. The average cerebrospinal fluid (CSF) pressure before and after the procedure was 352.50 mmH_2_O and 199.38 mmH_2_O, respectively. One patient suffered cerebral edema after the operation, which was confirmed by CTV to be due to thrombosis within the stent. As a result, this patient experienced cerebral infarction and secondary epilepsy. One patient experienced intervention failure, but improved after lumbo‐peritoneal shunt (LPS) as revision surgery. Another patient experienced a short‐term cough‐related headache. No other complications were observed during or after the procedure (Table 2).

**TABLE 2:** Intervention and follow‐up outcome.

Patient	Stenting location	Procedural complication	Last Documented CSF pressure (mmH_2_O)	Last follow‐up (month)	Last follow‐up of major symptoms			
					Headache (HIT‐6)	Papilledema (Frisen score)	Other visual symptoms	Pulsatile tinnitus
1	R transverse sinus	None	200	47	40	Stage I	No	No
2	R transverse sinus	None	260	18.5	36	Stage I	Yes	No
3	R transverse sinus	None	210	13	36	Stage II	Yes	No
4	R transverse sinus	None	200	31.5	36	Stage II	Yes	No
5	R tran/sig	Yes	110	13	44	Stage I	Yes	No
6	R tran/sig	None	260	44	36	Stage I	Yes	No
7	R tran/sig	None	190	45	36	Stage I	No	No
8	R tran/sig	None	190	48	36	Stage II	Yes	No
9	R transverse sinus	None	215	55.5	38	Stage I	No	No
10	R transverse sinus	None	175	51	36	Stage I	No	Yes
11	R tran/sig	None	305	66.5	36	Stage II	Yes	No
12	R tran/sig	Yes	230	73	36	Stage II	Yes	No
13	R tran/sig	None	185	54.5	36	Stage II	No	No
14	R transverse sinus	None	150	51	36	Stage I	No	No
15	R transverse sinus	None	190	61.5	36	Stage I	No	No
16	R transverse sinus	None	120	24.5	36	Stage II	Yes	No

Abbreviations: CSF, cerebrospinal fluid; HIT‐6, Headache Impact Test‐6; L, left; R, right; tran/sig, transverse/sigmoid; tran/sig, transverse/sigmoid.

### 3.3 Outcomes and follow‐up

All the patients experienced improvement, stabilization, or resolution of their papilledema following the VSS procedure. After a median follow‐up of 47.5 months (range 13 to 73), all patients who suffered from headaches (8/8) saw improvement (Mean HIT‐6 score declined from 66.9 to 37.8), while 81.25% (13/16) of patients who had blurred vision saw improvement, 75% (3/4) of patients who had amaurosis saw improvement, and 50% (1/2) of patients who had pulsatile tinnitus saw improvement. All patients but one (15/16) experienced improvement in at least one symptom, although only 37.5% (6/16) experienced complete resolution of symptoms related to elevated ICP (Table 2). Unfortunately, three patients' visual acuity did not improve due to irreversible optic nerve damage prior to the intervention. One patient's symptoms worsened 2 years after the procedure and stent wall thrombosis was discovered on CTV. Following a three‐month course of repeated dural antiplatelet therapy, the patient experienced complete relief from her headaches, which have not worsened since then. Subsequent CTV examinations demonstrated gradual organization of the thrombosis.

## 4 DISCUSSION

IIH is an uncommon condition characterized by symptoms of increased intracranial pressure with no known cause. According to previous case studies, headache, visual impairments, and pulsatile tinnitus are the most frequently reported symptoms of IIH. In our cases, all patients had papilledema and visual disturbances, but only 50% experienced headaches, and only two patients reported pulsatile tinnitus. This may result in a bias, as headaches and tinnitus symptoms may not be given as much consideration by Chinese patients, while visual disturbances are more likely to cause significant distress and lead to seeking medical attention.

Studies in case series have suggested that weight plays a significant role in the development of idiopathic intracranial hypertension. Obesity, which is associated with increased intra‐abdominal and intrapleural pressure, and then impede the outflow of CSF and result in elevated ICP.^7^ However, the proportion of overweight or obese patients in our series (50%) is significantly lower compared to those reported in western countries (where it is ≥90%), indicating that other factors may contribute more significantly to the development of this condition in our patients.

Dural sinus stenosis was a condition reported in 30–93% of patients with IIH, most commonly affects the right transverse sinus or the junction between the transverse and sigmoid sinuses.^8^ The relationship between venous stenosis and elevated ICP remains a topic of debate, as it is unclear whether the venous narrowing is a cause or a consequence of increased ICP. One theory suggests that elevated ICP leads to secondary narrowing of the sinus lumen by extrinsic compression, which can be reversed by reducing CSF pressure or diverting it. Alternatively, intrinsic obstruction, such as arachnoid granulations or fibrous septa, could obstruct venous outflow and lead to increased CSF pressure. In this case, a pressure gradient across the stenosis would be present, and stent implantation would effectively lower elevated ICP.

Our understanding of IIH has undergone changes over time.^9^ Quincke reported the first cases of IIH in 1897, shortly after introducing lumbar puncture into medicine. The condition was named pseudotumor cerebri in 1904. In 1955, Foley introduced the term “benign intracranial hypertension,” but the high incidence of visual loss in reports from the 1980s demonstrated that the term “benign” is no longer appropriate. Therefore, it is more accurately referred to as “idiopathic intracranial hypertension.” Currently, it has been discovered that a portion of IIH is caused by venous sinus stenosis, leading to obstructed CSF outflow. These patients theoretically should not be classified as having IIH, although they are still referred to as such.

Treatment options for IIH vary from conservative to surgical interventions.^10^ The conservative options include weight loss, medication (such as acetazolamide and topiramate), and lumbar punctures. For the majority of IIH patients, a combination of medical treatment and weight reduction is sufficient to alleviate symptoms and prevent progression. However, approximately one‐fourth of patients may require surgical intervention if they are facing visual loss or persistent severe headaches. In the past, patients who did not respond to medical treatment were typically referred for optic nerve sheath fenestration (ONSF) or CSF diversion procedures (such as VPS or LPS).^11^ In recent years, VSS has emerged as a potential superior alternative intervention. Although VPS or LPS have a longer history of treating IIH, theoretically, VSS is more appropriate for IIH caused by venous sinus stenosis because stent implantation belongs to etiological treatment.

A review of 143 published patients showed that 88% of patients reported improvement in headaches, while 97% reported resolution of papilledema and 87% reported improvement or resolution of visual symptoms.^12^ A single‐center experience of 52 patients with IIH who received VSS found that stent placement immediately eliminated the pressure gradient, resulting in significant improvement of symptoms and resolution of papilledema.^13^ The relationship between the pressure gradient of venous sinus stenosis and the success of VSS in IIH appears to exist.^14^ Our cases also showed similar efficacy, with 100% resolution, improvement, or stabilization of their papilledema, 100% improvement in headaches, 81.25% improvement in blurred vision, and 75% improvement in amaurosis.

Some experts believe that it is important to distinguish between intrinsic and extrinsic compression of the venous sinuses in order to make informed treatment decisions, such as shunt surgery for reversible stenosis (extrinsic compression) and stent placement for fixed stenosis (intrinsic compression). However, a study by Lenck et al. showed that stenting of the lateral sinus stenosis was an effective treatment for IIH symptoms regardless of the type of stenosis.^15^ All of our patients also experienced improvement in their symptoms after stent implantation. We tended to believe that the relationship between venous stenosis and elevated ICP is a vicious cycle, rather than a straightforward cause‐and‐effect relationship. Resolving venous stenosis, regardless of cause or consequence as it is, could help to reduce ICP and improve symptoms. The clear benefit in symptoms for patients with IIH who received stents highlights the role of lateral sinus stenosis in this condition, although the mechanisms controlling CSF drainage are not yet fully understood.

The complications of VSS are rare and usually associated with the angiographic procedure itself. Major complications include vessel perforation, subdural hematoma, subarachnoid or parenchymal hemorrhage, stent thrombosis, and restenosis.^16^ Ahmed et al.'s study reported two significant neurological complications, including one subdural hematoma caused by guidewire perforation and one complex hemorrhage, both of which resolved after craniotomy.^13^ In a meta‐analysis of 395 patients with follow‐up data on stenting outcomes (mean of 18.9 months), the stent survival and stent‐adjacent stenosis rates were 84% and 14%, respectively, with <2% of patients experiencing major neurological complications.^17^ In our study, only one patient suffered cerebral infarction with secondary epilepsy due to stent thrombosis, and fully recovered after symptomatic treatment. One patient experienced intervention failure, but improved after LPS as revision surgery. No other major complications were observed during the procedure.

Although many case series have demonstrated the effectiveness of the procedure, there are limited studies with long‐term follow‐up compared to ONSF and CSF diversion studies. A previous study found that 10.4% (5/48) of patients experienced in‐stent stenosis with a median follow‐up of 49 months by digital subtraction angiography (DSA) examination.^18^ These in‐stent stenosis was found at 22, 8, 18, 4, and 11 months after stent deployment. During our nearly 4‐year follow‐up period, only one patient experienced recurrence and stent wall thrombosis, which was confirmed 2 years later after stenting. The latest systematic review comparing different surgical treatments for IIH showed that VSS is the most promising treatment option given its remarkable results in headache resolution and visual improvement, low failure rates, and favorable complication profile.^4^

## 5 CONCLUSIONS

Venous sinus stenosis is a commonly observed condition in patients with IIH. For those who have failed MMT, we found that VSS is an effective treatment option with a favorable complication profile and low recurrence rate. Our findings reconfirm the long‐term efficacy of VSS in IIH patients with relatively low BMI compared to their western counterparts.

## FUNDING INFORMATION

This work was supported by the Science Technology Department of Zhejiang Province (GF19H090009) and Zhejiang Provincial Natural Science Foundation of China (LQ20H090007).

## CONFLICT OF INTEREST STATEMENT

None declared.

## Data Availability

The data that support the findings of this study are available from the corresponding author upon reasonable request.
